# The Nursing in Prison Infirmaries

**Published:** 1898-06-04

**Authors:** 


### The Institutional Workshop.

[We have lately published several articles dealing with prison management. These articles have borne upon their face the impress of being written with full knowledge of the subject; and, indeed, so patent has this been that we have been accused—and perhaps not altogether without reason—of having given the official side of the question. What is evident is that there ought to be no official side, and, so far as mere facts go, we do not think the officers of our prisons can fairly be charged with the slightest desire to put forward anything which is not literally correct. In human affairs, however, facts are not everything, and our prisons contain such a varied mixture of different types of humanity, the crimes are so different, the character of the criminals varies so much, the weight of their sentence falls so much more heavily on one than on another, that it is not to be wondered at that the aspect of the same facts should vary greatly according as they are looked at from a repression-of-crime point of view, or from that purely humanitarian standpoint which regards all men as brothers. We are, in fact, so fully conscious of the existence of another side to almost every question—especially to such as affect institutions which are under lock and key—that we have asked a lady, whom we know to have had exceptional opportunities for obtaining recent information regarding the treatment of the sick in prisons, to give us the result of her inquiries into this matter. There is no doubt that some of the statements which she makes are such as to give rise to some uneasiness; but, having asked her for her views, always recognising that they express that "other side" which lies behind every question, we do not hesitate to give them a publicity equal to that which we gave to the original articles.]

### THE NURSING IN PRISON INFIRMARIES.

By a Trained Nurse.

I.

Recently the prisoner has been occupying a good deal of public attention. Whether the methods of punishment are humane, or whether the recreations and appetites of persons under sentence are sufficiently considered and philanthropically dealt with, are questions over which much controversy has arisen. But no member rose from his place in the House of Commons to inquire into the nursing of the sick in our prison infirmaries; yet in this particular lies the weak spot of our prison system. While the care of the sick in our hospitals has reached a most desirable point, the treatment accorded the patients of our prison infirmaries belongs rather to the Middle Ages than to progressive modernism.

In the prison wards to-day one may see a very fair example of what was the type of nursing in the average hospital of 30 years ago, with this difference, that the prison wards are very clean. I was accorded a very courteous permission to go the round of the prisons, and a tour of the infirmaries of the leading gaols and convict prisons both in England and Ireland—viewing those with unprejudiced, though professional eyes—has led me to the conclusion that the management of the sick wards constitutes the only unsatisfactory feature of prison life. The average prisoner is extremely well off and comfortable. He lives in well warmed cells, is humanely treated, and, speaking generally, has a much better time than he has any right to expect.

Public sentiment is easily roused, but in the present instance the popular mind has shown a rare good sense in not rising to the sensational baits recently prepared as to the "refined cruelties" and "modern tortures" of the prison.

There is, however, a wide difference between the hale and hearty criminal with strength and disposition to prosecute his evil tendencies against social well-being and the same man brought low by rheumatic fever or pneumonia. In this condition he has the human right to ask for skilful care and proper nursing. And while I bear testimony, with pleasure, to the extreme kindness evidenced by the warders towards their sick prisoners, I must at the same time maintain that a sick person—however criminal—should be nursed by trained hands. And the trained nurse is unknown in the prison wards. In most prisons there are general sick wards—the number varying with the number of prisoners confined in the building—and some prisoners are cared for in separate cells. The separate cell patients are mostly those who are more seriously ill—the delirious and the noisy; and the patients in the general wards are placed under far better conditions than are those whose condition calls for a separate cell—for these latter with few exceptions are dark and unhygienic, and in some instances, as at Wormwood Scrubs, are below the ground level. So that the sick man's heritage of fresh air and sunlight is in some instances a structural impossibility.

The personal care, cleanliness, and disinfection of patients and surroundings, which in the case of our hospitals are matters of routine, are conspicuously absent in prison infirmaries, where the sick are in charge of women totally untrained and unskilled in these essentials of nursing. A prison warder is promoted to the infirmary—not as a question of fitness or experience in her work, but simply as a matter of routine and normal progression.

The type of woman put in charge of the wards of prison infirmaries is of an excellent type for service as warders, but from the point of view of sick nurse is necessarily a failure, for of the art of nursing these know nothing. I asked a prison governor in the event of a trained nurse being willing to enter the prison service as a warder, in the hope of ultimately gaining charge of a sick ward, whether such a nurse would be chosen for service in the infirmary? His answer was, "Certainly not. If she finally gravitated to the infirmary wards it would be a question of chance. There is no provision in the rules which would lead to the preference of such a warder for infirmary duty."

It must be quite understood that in this statement of matters as they exist in prison wards no possible reflection is intended on female warders. In my wanderings I have been impressed with much admiration of the woman warder, of her patience, self-control, and kindness. And I must confess to having thought many a time of the hardships inflicted on the warder by the popular admiration of the nurse-heroine, while a woman engaged in the arduous, the unselfish, and most trying work of prison supervision has never received one word of praise or recognition from the public.

The hospital nurse has many compensations for her hard labours in the wards—she receives some measure of gratitude and affection from her patients and their friends. There is much pleasant social intercourse in her daily life. The prison warder—though she be immensely kind and womanly, has little outlet for her sympathies; she certainly receives no affection or gratitude from the grim-visaged "submerged tenth" by whom she is for the most part surrounded. Her hours are long, her duties hard, and the weary monotony of her task, and the absence of any of those changes whose "lightsomeness" we all recognise, combine to make her life a very dreary one.

During a visit to Holloway Gaol, in the course of a conversation with one of the prison medical officers, he said that "in his opinion no trained nurse would accept office in a prison ward." But this is a mere unsupported statement, for the question has never been put to the test. The nurse has not been afforded an opportunity of working in such wards, so that prison infirmaries of to-day are, as were all the refuges of the sick in the dark ages, in the hands of women who have picked up some of the elements of nursing, but who are absolutely untrained in the professional sense of the word. All the officials with whom I discussed the question were quite unanimous in asserting that, even were the rules to be altered so as to admit of trained nurses being employed in prison infirmaries, such nurses "would not endure for a month the dulness and monotony of a prison ward." To anybody conversant with the trained nurse and her habits this statement would need much proof and demonstration. For the annals of nursing have never yet recorded work so disagreeable, dangerous, or monotonous that trained nurses have not been eager and ready to adopt it—in the cause of charity and humanity. Nurses who have not shrunk from small-pox, typhus, and lock wards, from cholera and plague epidemics, are not likely to find their courage fail them at the prospect of a prison ward! For the nurses of all ages have been of better fibre and stuff than to shrink from possible dulness when there is good work to be done. And in the prison wards there is good work to be done; and, though one hesitates to embark on prophecy, it is quite safe to predict that in a comparatively short time no prison ward will be found in the hands of untrained women.

